# Cultural heritage of the Qin-Shu Ancient Road in Shaanxi: Spatial distribution characteristics and influencing factors

**DOI:** 10.1371/journal.pone.0331676

**Published:** 2025-09-09

**Authors:** Kankan Li, Ziqiang Huang, Yinghui Pang, Sijie Chen, Yijing Fan, Menglei Yin, Chengyong Shi

**Affiliations:** College of Landscape Architecture and Art, Northwest Agriculture and Forestry University, Xianyang, China; Banaras Hindu University, INDIA

## Abstract

This study investigates the spatial and temporal distribution and the influencing factors of 579 cultural heritage sites along the Qin–Shu Ancient Road in Shaanxi Province, employing kernel density estimation, buffer analysis, and geographic detectors. Three key findings emerge: (1) The spatial pattern is characterized by a “line-belt-core” structure, with a belt-like aggregation along the Xi’an-Baoji-Hanzhong axis. Core concentrations are found in Xi’an (181 sites), Hanzhong (159 sites), and Ankang (122 sites), with secondary concentrations in Baoji (72 sites) and Shangluo (36 sites). The spatial distribution of heritage types—such as ancient road relics, traditional villages, historic architecture, cave temples and rock carvings, modern historic sites, and tomb complexes—is influenced by topography and human factors. (2) The spatiotemporal evolution occurs in three stages: “natural selection,” “technological breakthrough,” and “cultural adaptation.” It begins with linear valley distributions during the Prehistoric-Qin-Han period, expands through mountainous regions with plank-road engineering in the Wei-Jin-Sui-Tang period, and shifts towards settlements and tomb complexes from the Song to Qing dynasties, marking a transition from transport corridors to integrated cultural landscapes. (3) Natural factors—such as elevation, precipitation, slope, aspect, and proximity to hydrological networks—are the primary drivers, while anthropogenic factors, including ancient route alignment and regional GDP, have a secondary influence. Significant interactions were observed between elevation and aspect, elevation and precipitation, and between hydrological proximity, aspect, and precipitation. These findings offer both theoretical insights and practical guidance for heritage conservation planning and the development of regional cultural tourism along the Qin-Shu Ancient Road.

## 1. Introduction and research aim

Cultural heritage serves as a spatiotemporal coordinate in the process of human civilization, not only carrying the cumulative construction of historical memory but also acting as a material vehicle for the transmission of cultural genes [1]. Within the multi-ethnic and unified framework of Chinese civilization, linear cultural heritage, characterized by its connectivity across geographical barriers, provides a crucial perspective for studying civilizational interactions and cultural diffusion [2]. The Qin-Shu Ancient Road, a vital “southbound transportation artery” in ancient China, originated in the Neolithic period, expanded during the Qin and Han dynasties, and reached its peak in the Sui and Tang dynasties. This route system comprises multiple sub-routes, including Baoxie Road, Ziwu Road, Chencang Road, Tangluo Road, and Wuguan Road [3]. The heritage resources along these routes encompass physical cultural relics, such as ancient roads, passes, post stations, and inscriptions, making the Qin-Shu Ancient Road a “living specimen” for studying China’s ancient transportation civilization, frontier governance, and cultural exchanges [4].

However, under the dual pressures of rapid urbanization and global climate change, this heritage corridor faces significant threats. On one hand, geological disasters (with annual landslide displacement exceeding 15 cm) and hydrological changes (a 23% shift in the course of the Han River’s mainstream) are accelerating the deterioration of heritage sites [5]. On the other hand, infrastructure development has led to the fragmentation of heritage sites, while commercialization driven by tourism threatens their authenticity and integrity [6]. In this context, a critical academic challenge arises: how to quantitatively analyze the spatial differentiation patterns of these heritage sites and develop adaptive conservation strategies.

In recent years, GIS-based spatial analysis techniques have provided a new paradigm for the quantitative study of cultural heritage. Methods such as kernel density estimation (KDE), spatial autocorrelation analysis, and Geodetector models have proven effective in deciphering the spatial heterogeneity of heritage distribution and its driving mechanisms [7]. Existing research on cultural heritage spatiality primarily falls into three categories: spatial pattern analysis, spatiotemporal evolution mechanisms, and exploration of driving factors.

Analyzing the spatial distribution of cultural heritage is key to understanding its formation mechanisms. Recent studies employing KDE, spatial autocorrelation, and standard deviation ellipse methods have revealed distinct spatial distribution patterns. For instance, Liao Lanqin’s study [4] on the spatial distribution of intangible cultural heritage in the Yangtze River Basin found a significant correlation with river accessibility and economic factors. Verhagen & Jeneson [8] examined the spatial distribution of Roman road relics in the Netherlands, identifying key influences such as topography, military defense, and urban layout. Tim Williams [9] investigated the Silk Road heritage sites and proposed a conservation strategy based on GIS and the Landscape Heritage Approach. These studies highlight that cultural heritage distribution is shaped by both natural factors (topography, hydrology, climate) and socio-economic elements (transportation, policies, urbanization), emphasizing the necessity of an integrated, corridor-based management approach for linear cultural heritage.

Research on the spatiotemporal evolution of cultural heritage helps uncover historical distribution patterns and their driving forces. Sun Haiyan et al. [10] studied the evolution of Silk Road heritage sites, revealing a “concentration-followed-by-diffusion” pattern influenced by political and economic shifts. Zhao Jianming et al. [11] analyzed cultural heritage evolution in the Yellow River Basin, demonstrating the significant impact of hydrological changes on heritage site migration. Graham Fairclough (2018) [12] explored European cultural heritage routes, such as the Camino de Santiago, introducing the concept of “Dynamic Conservation of Cultural Landscapes” to emphasize heritage evolution. Zapata et al. (2020) [13] investigated the historical evolution of the Inca Road system, identifying the role of mountainous terrain and colonial road modifications, and proposed an integrated cultural landscape conservation strategy.

Moreover, cultural heritage distribution is influenced not only by natural constraints but also by socio-economic dynamics, policy frameworks, and transportation accessibility. Xu, X. W. et al. [14] analyzed the spatiotemporal distribution of historical cultural heritage in Tibetan regions using information entropy, revealing its “dispersed-aggregated” fluctuation trend, intergenerational differences, and cross-era correlations. Pang, L. et al. [15] analyzed the spatiotemporal distribution of intangible cultural heritage in the Beijing-Tianjin-Hebei region, revealing its concentration in the southern-central areas, the dominant influence of economic development and social culture, and the relatively minor impact of the physical environment. Capello & Nijkamp (2019) [16] developed a cultural value assessment model for the Appian Way, demonstrating the impact of economic vitality, tourism development, and local governance on heritage conservation.

Existing research on the Qin-Shu Ancient Road primarily focuses on historical and cultural aspects, including textual studies on historical background [17–19], route mapping [20–24], and archaeological discoveries along the route [25]. Researchers such as Yan Zhijun have conducted in-depth studies on route distribution and published extensive works on the ancient transportation system [26]. Since the early 21st century, with the initiation of the Qin-Shu Ancient Road’s World Heritage nomination process, studies have expanded to include route conservation strategies [27–29] and tourism planning [30–32]. Despite these advancements, significant research gaps remain. First, existing studies are fragmented, often focusing on individual route verifications (e.g., Li Zhiqin’s textual examination of Baoxie Road mileage [33]), lacking a systematic spatial analysis of the overall network. Second, methodological innovation is limited—traditional research predominantly relies on historical textual analysis and archaeological typology (e.g., Yan Zhijun’s route reconstruction study [26]), with few applications of spatial econometric models (e.g., Ripley’s K function and Geodetector models) to uncover underlying spatial distribution mechanisms. Third, theoretical explanations are insufficient, as most studies remain descriptive, failing to elucidate the coupled interactions between natural and human factors, such as the interplay of topographical constraints and military defense strategies. This disjointed theoretical-methodological-practical framework has resulted in a conservation approach overly focused on physical site restoration while neglecting systematic corridor management.

To address these gaps, this study focuses on the Shaanxi section of the Qin-Shu Ancient Road, integrating multi-source spatiotemporal data (historical documents, remote sensing imagery, and field surveys) to establish a comprehensive “pattern identification–process interpretation–mechanism exploration” research framework. Specifically, (1) kernel density estimation and standard deviation ellipse methods are employed to quantitatively characterize heritage clustering patterns and directional trends; (2) historical map overlay analysis and centroid migration models are applied to reconstruct spatiotemporal evolution trajectories; and (3) Geodetector models are utilized to assess the driving forces of natural geographic factors (elevation, slope, river buffers, aspect, precipitation, temperature, soil type) and human influence factors (GDP, ancient transportation, ethnic culture). This study aims to advance GIS applications in heritage conservation methodology and provide scientific support for the preservation of the Qin-Shu Ancient Road’s cultural heritage.

## 2. Materials and methods

### 2.1. Study area

**Fig 1:**
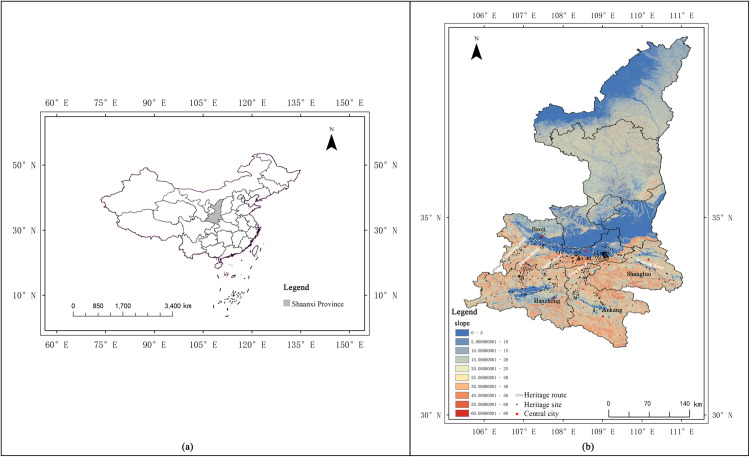
Geographic location. **(a)** Map of China. **(b)** Spatial Distribution of Qin-Shu Ancient Road Cultural Heritage in Shaanxi Province. This figure was generated using ArcGIS 10.2 and is intended for illustrative purposes only.

The Qin–Shu Ancient Road extends from Xi’an in Shaanxi Province to Chengdu in Sichuan Province, spanning approximately 4,000 km, with about 70% of the extant segments located within Shaanxi. Therefore, this study focuses on the Shaanxi section of the route, covering five prefecture‐level cities (Xi’an, Baoji, Shangluo, Ankang, and Hanzhong) across 32 counties. We investigated five major road branches—Bao–Xie, Zi–Wu, Chen–Cang, Tang–Luo, and Wu–Guan—as well as 579 related cultural heritage sites (Fig 1). The selection was based on documented historical records directly linking each site to these ancient road routes. Only those culturally, historically, and functionally relevant locations within Shaanxi’s administrative boundaries were included in the final analysis.

### 2.2. Data sources and processing

The data for the 579 cultural heritage sites along the Qin-Shu Ancient Road in Shaanxi primarily come from the *Shaanxi Provincial Immovable Cultural Relics List (2022 Edition)*, the Third National Cultural Relics Census (2007–2011), and the “Cultural Heritage of the Qin-Shu Ancient Road” dataset. Additional information was obtained from the Shaanxi Provincial Cultural Heritage Bureau website (https://wwj.shaanxi.gov.cn/) and the Shaanxi Provincial Cultural Heritage Institute (http://www.sxswyy.com/).

To ensure spatial alignment accuracy of the ancient road network, vector calibration was performed using the “Historical Atlas of China” and the “Research on the Ancient Road Network of Shaanxi.” ArcGIS topology tools were used to correct coordinate deviations and verify the alignment precision between historical maps and modern administrative boundaries. The spatial data includes elevation (30-meter resolution), slope (1-kilometer resolution), and aspect (1-kilometer resolution), sourced from the Geospatial Data Cloud (https://www.gscloud.cn/) and Google Maps. Climate data (precipitation and temperature) was extracted from the “3000-Year Chinese Meteorological Records” available at the China Meteorological Data Network (https://data.cma.cn/). Socio-economic data (GDP) is sourced from the “2022 Shaanxi Statistical Yearbook” (https://tjj.shaanxi.gov.cn/). River system data is obtained from the National Geographic Information Resource Directory Service System (https://www.webmap.cn/), and soil data is from the China Soil Database (http://vdb3.soil.csdb.cn/). Administrative boundary data were downloaded from the National Geomatics Center of China (http://bzdt.ch.mnr.gov.cn/).

To ensure data reliability, this study includes only heritage sites with clearly defined archaeological dates (e.g., C14 radiocarbon dating or cross-referenced with historical documents) or those designated as provincial-level or higher cultural heritage protection units, excluding sites with unclear or disputed dates. The classification of heritage sites follows the “Chinese Cultural Heritage Protection Standards” (2015) and the “Cultural Routes Convention” (ICOMOS, 2008). All data sources are listed in Table 1.

**Table 1:** Summary of Data Sources.

Data Type	Source Name	Source Website	Notes
Immovable Cultural Relics Data	Shaanxi Provincial Immovable Cultural Relics List (2022 Edition)	https://wwj.shaanxi.gov.cn/	Main dataset for 579 heritage sites
Heritage Survey Data	Sixth National Cultural Relics Survey (2007–2011)	http://www.ncha.gov.cn/	Supplemental data for heritage sites
Heritage Date Information	Shaanxi Historical Atlas	http://wwj.shaanxi.gov.cn/	Provides basis for heritage site date classification
Ancient Road Network	Historical Atlas of China, Research on the Ancient Road Network of Shaanxi	https://www.sinomaps.com/	Vector calibration of road paths
Administrative Boundaries	National Geomatics Center of China	http://bzdt.ch.mnr.gov.cn/	Delineates study area and overlays socio-economic data
Elevation, Slope, Aspect	Geospatial Data Cloud	https://www.gscloud.cn/	30-meter resolution elevation, 1-kilometer resolution slope/aspect data
River System	National Geographic Information Resource Directory Service System	https://www.webmap.cn/	Analyzes the relationship between cultural heritage and river systems
Economic Data	2022 Shaanxi Statistical Yearbook	https://tjj.shaanxi.gov.cn/	Provides GDP data
Precipitation and Temperature Data	3000-Year Chinese Meteorological Records	https://data.cma.cn/	Historical climate data for environmental analysis
Soil Data	China Soil Database	http://vdb3.soil.csdb.cn/	Soil data for environmental analysis

Geographic coordinates for the heritage sites were extracted via Google Maps and imported into ArcGIS 10.2 for spatial analysis. All heritage sites were projected to the WGS-84 coordinate system, and a standardized database was constructed to support subsequent analyses, including kernel density estimation, nearest neighbor indices, and geographic detector models.

### 2.3. Research methods

Based on the collected data, ArcGIS 10.2 software was used to analyze the spatial–temporal distribution of cultural heritage along the Qin–Shu Ancient Road in Shaanxi Province and to identify its influencing factors, using methods such as the nearest neighbor index, kernel density analysis, buffer analysis, and geographic detectors.

**Ethics statement**

All participants provided written informed consent to take part in this research. Written informed consent was also obtained from the individual(s) for the publication of any potentially identifiable images or data included in this article.

#### 2.3.1. nearest neighbor index.

To examine the spatial distribution of heritage sites along the Qin–Shu Ancient Road, the nearest neighbor index was applied. The nearest neighbor index is defined as the ratio of the actual nearest neighbor distance to the expected nearest neighbor distance, and it can be classified into three types: uniform distribution, clustered distribution, and dispersed distribution. The calculation method is as follows [34]:


$$\overset{―}{r_{e}}=\frac{1}{2\sqrt{\frac{n}{A}}}$$ (1)

$$R=\frac{\overset{―}{r_{i}}}{\overset{―}{r_{e}}}$$ (2)

In the formula, $\overset{―}{{\text{r}}_{\text{e}}}$ represents the expected nearest neighbor distance; n is the number of point features in the study area; A is the area of the study area; $\overset{―}{r_{i}}$ represents the actual nearest neighbor distance; R is the nearest neighbor index. If R < 1, the distribution type is clustered; if R = 1, the distribution type is dispersed; if R > 1, the distribution type is uniform.

#### 2.3.2. Kernel density analysis.

The application of kernel density analysis can visually depict the spatial distribution characteristics of point features, thus providing a means to examine the central areas of cultural heritage distribution along the Qin-Shu Ancient Road in Shaanxi. The formula is as follows [35]:


$$f(x)=\frac{1}{nh}\sum_{i=1}^{n}k\left(\frac{x-x_{i}}{h}\right)$$ (3)

In the formula, f(x)represents the kernel density; n is the number of observed point features; h is the search radius; $x-x_{i}$ denotes the distance from the estimated point x to sample point $x_{i}$. A higher f(x) indicates higher density of point features, while a lower f(x) indicates lower density of point features.

#### 2.3.3. Standard deviation ellipse.

The Standard Deviation Ellipse (SDE) method is used to analyze the spatial distribution characteristics of cultural heritage sites along the Qin-Shu Ancient Road. This method calculates the deviation of the heritage sites relative to the centroid, revealing the distribution direction and spatial evolution trends. The formula is as follows [36]:

Standard Deviation formula:


$$s_{x}=\sqrt{\frac{1}{n}\sum_{i=1}^{n}({\hat{x}}_{i}-\bar{x}{)}^{2}},\text{\textbackslash{} \textbackslash{} }s_{y}=\sqrt{\frac{1}{n}\sum_{i=1}^{n}({\hat{y}}_{i}-\bar{y}{)}^{2}}$$ (4)

Where ${\hat{x}}_{i}$ and ${\hat{y}}_{i}$ are the coordinates of the i-th heritage site, and $\bar{x}$ and $\bar{y}$ are the mean coordinates.

Standard Deviation Ellipse formula:


$$Tan\theta =\sqrt{\left(\sum_{i=1}^{n}{\hat{x}}_{i}^{2}-\sum_{i=1}^{n}{\hat{y}}_{i}^{2}\right)+\sqrt{{\left(\sum_{i=1}^{n}{\hat{x}}_{i}^{2}-\sum_{i=1}^{n}{\hat{y}}_{i}^{2}\right)}^{2}+4{\left({\sum_{i=1}^{n}\hat{x}}_{i}{\hat{y}}_{i}\right)}^{2}}}$$ (5)

In this study, the SDE method is employed to reveal the spatial distribution and spatiotemporal evolution of cultural heritage sites along the Qin-Shu Ancient Road, providing insights into the clustering characteristics and their relationship with natural and socio-cultural factors.

#### 2.3.4. Geographical detector.

To investigate the spatial heterogeneity and driving mechanisms of the spatial distribution density of the Qin–Shu Ancient Road cultural heritage in Shaanxi (dependent variable Y), we selected natural geographic factors (e.g., altitude, slope, annual precipitation), historical factors (distance to ancient road), and socioeconomic factors (GDP) as independent variables X. We applied the Differentiation Detector to assess the explanatory power of each single factor on Y’s spatial variance and the Interaction Detector to examine pairwise interaction effects on Y.

All variables were classified into discrete strata in ArcGIS using the natural breaks method. The q-statistic for each single factor is calculated by [37–39]:


$$q=1-\frac{\sum_{h=1}^{L}N_{h}\sigma_{h}^{2}}{N\sigma^{2}}$$ (6)

where h = 1, 2, …, L denotes the stratum index; $N_{h}$ and $\sigma_{h}^{\text{2}}$ are the number of units and variance within stratum h; N and $\sigma^{\text{2}}$ are the total number of units and overall variance. q ∈ [0,1], and a higher q indicates stronger explanatory power for Y’s spatial differentiation.

For any two factors $X_{i}$ and $X_{j}$, the interaction q($X_{i}$ ∩ $X_{j}$) is compared with individual q-values to determine the interaction type (Table 2) [39].

**Table 2:** Types of Interaction Factor Detection.

Types of Interaction; Criteria for Determination	Types of Interaction; Criteria for Determination
Nonlinear weakening	q($X_{i}\text{\textbackslash{} }$∩ $X_{j}$) <Min(q($X_{i}$),q($X_{j}$))
Single-factor nonlinear weakening	min(q($X_{i}$), q($X_{j}$)) < q($X_{i}$ ∩ $X_{j}$) <max(q($X_{i}$), q($X_{j}$))
Two-factor enhancement	q($X_{i}$ ∩ $X_{j}$)> max(q($X_{i}$), q($X_{j}$))
Independence	q($X_{i}$ ∩ $X_{j}$) = q($X_{i}$) + q($X_{j}$)
Nonlinear enhancement	q($X_{i}$ ∩ $X_{j}$) > q($X_{i}$) + q($X_{j}$)

## 3. Results

### 3.1. Spatial distribution characteristics

**Fig 2:**
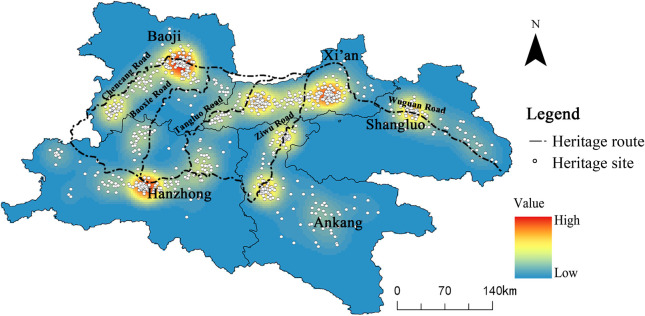
Qin-Shu Ancient Road Cultural Heritage Distribution Map. This figure was generated using ArcGIS 10.2 and is intended for illustrative purposes only.

According to statistics, there are 579 heritage sites related to the Qin–Shu Ancient Road (Fig 2), which can be classified into seven main types (Table 3). Among these, ancient road sites (n = 156, 26.9%) and ancient road remains (n = 131, 22.6%) together account for 49.9%, forming the core of the heritage. This confirms the primary function of the Qin–Shu Ancient Road as a transportation route (Ripley’s *L* index > 4.32, *p* < 0.01). Ancient architecture (n = 84, 14.5%) and traditional villages (n = 79, 13.6%) reflect the dense distribution of settlements and religious buildings along the route, with their spatial distribution being highly correlated with the ancient road’s post station system. Although fewer in number, ancient tombs (n = 40, 6.9%), modern historical sites (n = 50, 8.6%), and grottoes and stone carvings (n = 39, 6.7%) reflect the diverse functions of the Qin–Shu Ancient Road in burial culture, modern military activities, and religious dissemination.

**Table 3:** Statistical Analysis of the Number of Cultural Heritage Sites along Different Types of Qin-Shu Ancient Roads in Shaanxi Province.

Type of Cultural Heritage	Count
Ancient Road Site	156
Ancient Road Relics	131
Traditional Villages	79
Ancient Architecture	84
Cave Temples and Stone Carvings	39
Ancient Tombs	40
Modern-Era Historic Sites and Representative Buildings	50
Total	579

The heritage sites are primarily concentrated in five cities in southern Shaanxi, spanning 32 counties. Among these, Xi’an, Hanzhong, and Ankang represent the primary concentration areas, with 181, 159, and 122 heritage sites, respectively, accounting for 32.8%, 28.0%, and 21.1% of the total number in the province. Baoji and Shangluo are secondary concentration areas, with 72 and 36 heritage sites located in these regions.

The nearest neighbor index analysis of the Qin–Shu Ancient Road cultural heritage (Table 4) reveals significant differences in the spatial distribution patterns of various heritage types. Ancient road sites (R = 0.71), ancient road remains (R = 0.63), traditional villages (R = 0.69), important modern historical sites, representative buildings (R = 0.76), and grotto temples and stone carvings (R = 0.55) all exhibit clustered distribution patterns. The Z-values for these sites are significantly less than zero. This indicates that these heritage types are primarily concentrated in mountain and river valley areas accessible by transportation, closely associated with the historical transportation corridors of the Qin–Shu Ancient Road. In contrast, the R values for ancient architecture (R = 1.03) and ancient tombs (R = 1.21) exhibit a random distribution, reflecting their diverse historical functions and site selection criteria. Specifically, the location of ancient tombs may have been influenced by cultural and religious customs, often located in more isolated areas.

**Table 4:** Nearest Neighbor Index of Different Types of Cultural Heritage along the Qin-Shu Ancient Road.

Type	Average Nearest Neighbor Distance (km)	Expected Nearest Neighbor Distance (km)	R	P	Z	Distribution Model
Ancient Road Relics	17.49	24.63	0.71	0	−4.193	Clumped
Ancient Road Sites	27.43	43.53	0.63	0	−3.753	Clumped
Ancient Architecture	43.56	42.29	1.03	0.003	0.215	Random Distribution
Traditional Villages	31.09	45.05	0.69	0	−3.482	Clumped
Ancient Tombs	28.35	20.10	1.21	0	0.237	Random Distribution
Modern Important Historical Sites and Representative Buildings	75.41	99.22	0.76	0.765	−2.157	Clumped
Cave Temples and Stone Carvings	45.31	82.38	0.55	0.473	−3.376	Clumped

**Fig 3:**
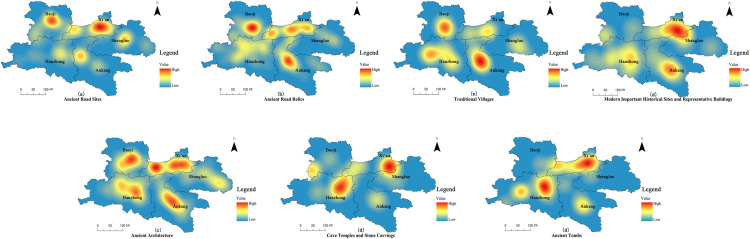
Kernel density analysis of different types of Qin-Shu Ancient Road cultural heritage. This figure was generated using ArcGIS 10.2 and is intended for illustrative purposes only.

Based on the kernel-density maps for Xi’an, Hanzhong, Ankang, Baoji, and Shangluo (Fig 3), the spatial distribution of each heritage type can be characterized as follows:

1 Ancient Road Sites

They form a linear, high-density corridor extending from Xi’an through Baoji to the Hanzhong Basin. Prominent hotspots in the Xi’an metropolitan area, northeastern Baoji, and central Hanzhong indicate that the main trunk of the Qin–Shu route and its auxiliary branches are most intensively preserved along this axis.

2 Ancient Road Relics

Similarly concentrated along the primary corridor, the remains show broader dispersion toward Ankang. Secondary hotspots in southwestern Ankang and southern Hanzhong suggest that roadside facilities and ancillary structures were particularly abundant at these nodal points.

3 Traditional Villages

The highest densities occur in the central Hanzhong Basin and northern Ankang, with subsidiary clusters near Xi’an and Baoji. Their distribution along valley floors and gentle slopes reflects favorable agro-ecological conditions and a close association with the historical post-station network.

4 Ancient Architecture

A pronounced core hotspot centers on Xi’an and its environs, with smaller, island-like concentrations in central Baoji and sporadic clusters in Hanzhong. This pattern underscores the primacy of Xi’an as a political–cultural center while reflecting diverse siting criteria in both mountainous and valley settings.

5 Cave Temples and Stone Carvings

Major hotspots occur in the northern Ankang mountains and southwestern Hanzhong, with minor secondary clusters in northern Shaanxi (Shaanbei). These sites are preferentially located on steep cliffs and isolated karst outcrops, consistent with their function as focal points of Buddhist and Daoist practice.

6 Modern-Era Historic Sites and Representative Buildings

Density peaks in Xi’an and Ankang, with additional moderate concentrations in Hanzhong and Baoji. The persistence of clustering along the ancient corridor reflects the continued strategic, administrative, and religious significance of these locales into the modern era.

7 Ancient Tombs

Tombs are relatively evenly dispersed, with low-intensity hotspots in Xi’an, Hanzhong, and Ankang. The mixed pattern of scattered points and short, linear bands indicates that burial siting was governed by both local funerary customs and terrain constraints.

### 3.2. Temporal and spatial pattern evolution

The Qin–Shu Ancient Road traces its origins to the Neolithic period, when rudimentary stone-rutted paths and hunter–gatherer trails first began to take shape (ca. 10,000–2000 BCE) [40,41]. It entered its founding phase during the Xia–Shang–Zhou dynasties (ca. 2100–221 BCE), as evidenced by Bronze Age trade routes and inter-tribal exchange records [42]. From the Warring States through the Qin–Han and Wei–Jin periods (ca. 475 BCE–420 CE), the construction of plank roads and beacon-fire relay stations, together with the establishment of an official courier system, marked its development into a structured, state-controlled network [43]. This trajectory culminated in the Tang–Song heyday (581–1279 CE), when an extensive road system—documented in epigraphic inscriptions and formal station registers—facilitated imperial administration and commerce [40]. During the Yuan–Ming–Qing transition and subsequent development stage (1271–1912 CE), the ancient road left diverse material legacies—adapted stations, roadside temples, official waystations, and upgraded bridges—that prefigured the shift toward early modern transport rather than constituting a modern highway system [44]. Although portions of the roadbed were modernized in the early Republican period, these works largely overlaid new highway alignments without substantially altering the ancient road’s form or usage context and did not constitute independently assessable heritage. Accordingly, our five-stage framework—Neolithic nascent, Xia–Shang–Zhou founding, Warring States–Qin–Han–Wei–Jin development, Tang–Song prosperity, and Yuan–Ming–Qing transformation—provides a coherent, evidence-based periodization of the Qin–Shu Ancient Road’s long-term cultural evolution.

**Fig 4:**
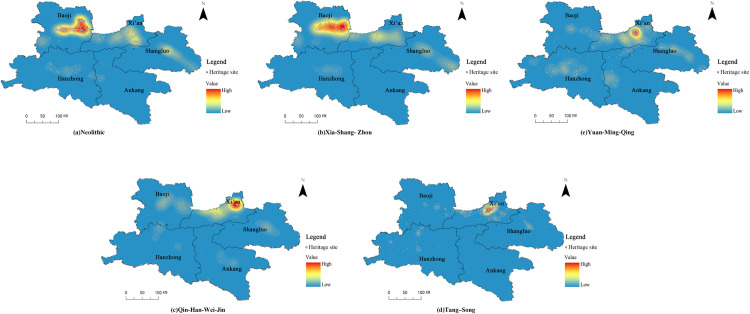
Kernel density analysis of Qin-Shu Ancient Road cultural heritage across different historical periods. This figure was generated using ArcGIS 10.2 and is intended for illustrative purposes only.

**Fig 5:**
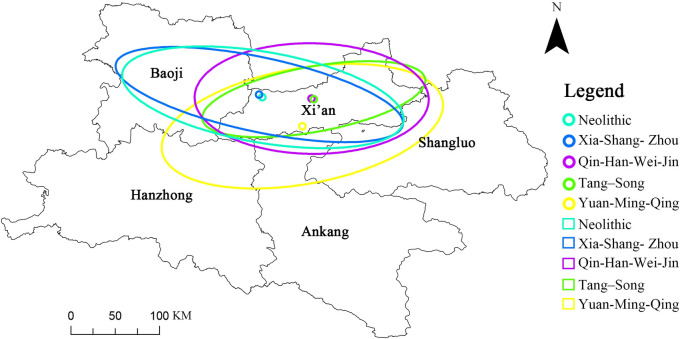
Centroid and standard deviation ellipse of the Qin-Shu Ancient Road heritage. This figure was generated using ArcGIS 10.2 and is intended for illustrative purposes only.

Using the Spatial Analyst extension of ArcGIS 10.2, we applied the Kernel Density tool to temporally segmented sets of 579 Qin–Shu Ancient Road heritage points, employing a fixed search radius of 10 km and a 1 km × 1 km output grid to produce stage-specific density surfaces that reveal clustering patterns across plains, valleys, and corridor alignments (Fig 4). We then used the Directional Distribution (Standard Deviational Ellipse) tool to fit weighted ellipses to each period’s heritage coordinates, quantifying east–west and north–south biases and overall spatial dispersion, thereby tracing a shift in the center of gravity from the southeast toward the southwest over time (Fig 5). Furthermore, the spatiotemporal characteristics of cultural-heritage distribution across historical periods are summarized in Table 5, which reports the number and spatial spread of heritage sites across distinct dynastic stages in Shaanxi Province.

**Table 5:** Distribution of Cultural Heritage Sites by Historical Period.

Historical Period	Total	Ancient Road Sites	Ancient Road Relics	Traditional Villages	Ancient Architecture	Stone Riches & Carvings	Ancient Tombs	Modern Important Historical and Cultural Buildings
Neolithic nascent	12	9	3	0	0	0	0	0
Xia–Shang–Zhou	20	11	6	0	0	0	3	0
Qin–Han–Wei–Jin	87	23	43	3	7	2	9	0
Tang–Song	311	60	123	20	69	19	20	0
Yuan-Ming Qing	149	14	24	10	61	3	5	32
Total	579	117	199	33	137	24	37	32

1. Neolithic and Xia–Shang–Western Zhou. With only 12 (Neolithic) and 20 (Xia–Shang–Western Zhou) sites, heritage is sparse and dominated by road alignments and tombs. Kernel-density hotspots concentrate on the Baoji–Zhouyuan plain, reflecting early pathway and settlement dependencies on topography and water resources.
2. Qin–Han, Wei–Jin, and Sui–Tang–Five Dynasties. Site counts surge from 87 to 311, with road sites and remains comprising a substantial majority. Density patterns extend from the Guanzhong heartland into the Shu corridor, and the ellipse center shifts southeastward by up to 0.48°in longitude, mirroring political–economic consolidation on the Guanzhong Plain and the expansion of the relay-station network and Buddhist–Daoist architecture.
3. Yuan–Ming–Qing. Although the total number of sites decreased to 149, the proportion of ancient architecture and modern historical buildings rose markedly; density exhibits a bimodal focus in the Hanzhong–Shangluo valleys, and the center of gravity moves southwestward with a north–south dispersion of 0.40°, indicating attenuation of the ancient corridor’s nodal function alongside persistent evolution of regional religious and vernacular cultural landscapes.

### 3.3. driving factors

The selection of influencing factors was based on the unique characteristics of the Qin–Shu Ancient Road cultural heritage in Shaanxi, a linear historical transportation route spanning multiple geomorphological zones. Drawing on cultural geography and heritage studies, we prioritized natural geographic variables (e.g., altitude, slope, annual precipitation) that may affect the distribution, accessibility, and preservation of heritage sites along the route. We also considered humanistic factors—including historical factors (e.g., distance to the ancient road, reflecting historical accessibility and site density) and socio-economic factors (e.g., regional GDP as a proxy for contemporary development pressures or conservation investments). These selections were informed by existing literature on the spatial–temporal distribution of cultural heritage [45–48] and validated through consultation with regional archaeology and heritage conservation experts to ensure alignment with local conditions. Data for each variable were obtained from authoritative sources, as detailed in Table 1. Preliminary exploratory analyses (e.g., distribution mapping and simple correlation tests) indicated that these factors capture the primary mechanisms influencing heritage distribution. Based on this, we finalized the set of key factors for analysis and employed the GeoDetector model to quantitatively assess the explanatory power of individual factors and the pairwise interactions among them, providing deeper insights into their combined effects on the spatial heterogeneity of heritage distribution.

#### 3.3.1. Physical Factors.

(1) **Altitude**

**Fig 6:**
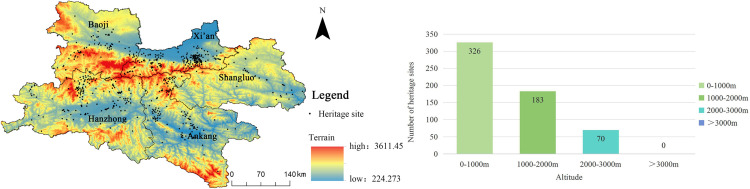
The relationship between the distribution of Qin-Shu Ancient Road heritage and the elevation. This figure was generated using ArcGIS 10.2 and is intended for illustrative purposes only.

To explore the relationship between the topography of Shaanxi and the distribution of heritage sites along the Qin–Shu Ancient Road, a spatial overlay analysis was conducted using elevation data for Shaanxi and the heritage distribution map of the Qin–Shu Ancient Road (Fig 6). The results reveal significant unevenness in the elevation distribution of these heritage sites, with a notable negative correlation to the elevation gradient (q = 0.85, p < 0.01). In low-altitude areas (<1000 meters), 326 heritage sites are located, accounting for 86.2% of the total, primarily concentrated in the Guanzhong Plain (Xi’an, Baoji) and the Hanjiang Valley (Hanzhong, Ankang). These regions, with an annual average temperature of 12–15°C and annual precipitation of 600–800 mm, provide favorable conditions for agricultural development. The flat terrain in these regions facilitated the establishment of ancient settlements and transportation hubs. In the mid- to high-altitude areas (1000–2000 meters), 183 heritage sites (11.9% of the total) are located, with 68% of these being postal station remains. The distribution in this zone is largely constrained by the topography of the Qinling Mountains. In high-altitude areas (>2000 meters), the number of heritage sites drops significantly, with only 70 sites (1.8% of the total), most of which are concentrated in military passes such as Dasanguan. Harsh climatic conditions and complex terrain significantly limit the distribution of heritage sites in these high-altitude regions.

(2) **River**

**Fig 7:**
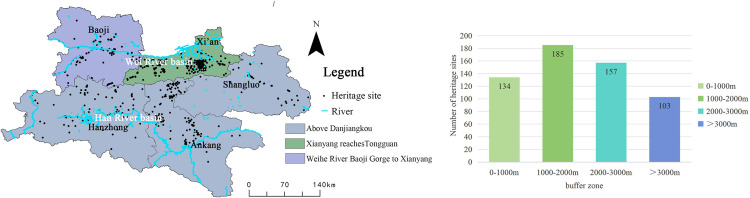
The relationship between the distribution of cultural heritage of Qin-Shu ancient road and rivers. This figure was generated using ArcGIS 10.2 and is intended for illustrative purposes only.

The Shaanxi Qin–Shu Ancient Road crosses multiple rivers, including the Yellow River, Yangtze River, Weihe River, Jialing River, and Hanjiang River. Based on river geography data for the Shaanxi region and the geographical information of the Shaanxi Qin–Shu Ancient Road heritage sites, a river buffer zone analysis was performed using the Shaanxi river data (Fig 7). Buffer radii of 1000 m, 2000 m, and 3000 m were designed. The results show that rivers have a decisive impact on the distribution of heritage sites (q = 1.00, p < 0.001), demonstrating both a water proximity clustering effect and watershed differentiation. The proximity clustering effect is reflected in the fact that 82% of heritage sites are located within the 3000 m river buffer zone. The 1000 m buffer zone accounts for 29.7% (134 sites), reflecting the early human settlement logic of “settling near water.” The watershed differentiation effect is demonstrated by the significantly higher heritage site density on the northern bank of the Hanjiang River (4.2 sites/100 km^2^) compared to the southern bank (2.1 sites/100 km^2^), which is directly related to the density of tributaries on the northern bank (0.85 km/km^2^) and the area of alluvial plains (63% of the area). Similarly, the concentration of heritage sites on the southern bank of the Weihe River in the Guanzhong region (e.g., the starting point of the Ziwudao) also confirms the guiding role of rivers in shaping transportation corridors.

(3) **Slope**

**Fig 8:**
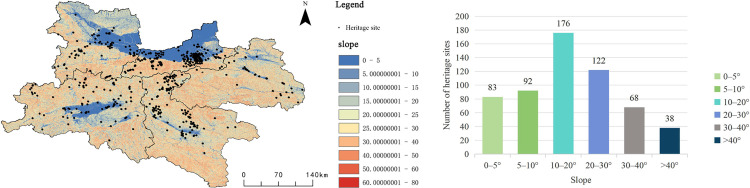
Relationship between Qin–Shu Ancient Road Cultural Heritage and Slope Gradient. This figure was generated using ArcGIS 10.2 and is intended for illustrative purposes only.

By extracting and analyzing Digital Elevation Model (DEM) data, slope information for the region was obtained, which served as the basis for investigating the impact of slope distribution on the spatial pattern of heritage sites (Fig 8). The results indicate that areas with slopes ranging from 10° to 20° contain 176 heritage sites, accounting for 46.6% of the total, making this the most densely distributed interval. Areas with slopes of 20°–30° comprise 122 heritage sites, representing 32.3% of the total. These moderate slope zones generally offer both suitability and defensive advantages, thus providing favorable natural conditions for early human migration, settlement selection, and the construction of transportation routes.

High-slope areas with gradients greater than 40° contain only 38 heritage sites, accounting for 10.1%, indicating that steep terrain significantly restricts transportation accessibility and construction feasibility, thereby reducing the density of heritage sites. Regions with gentle slopes (0°–5°) contain 83 heritage sites, or 11.0% of the total. Although gentle slopes are conducive to agricultural activities and settlement development, such areas are often limited in terms of strategic defense and resource diversity, and subsequent urban expansion and land development may have altered the original distribution patterns of heritage sites.

(4) **Aspect**

**Fig 9:**
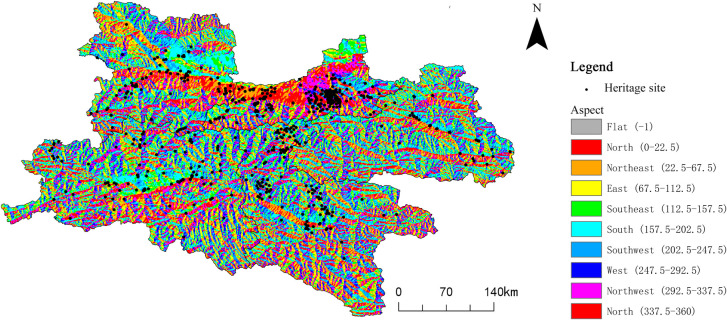
Relationship between Qin–Shu Ancient Road Cultural Heritage and Slope Aspect. This figure was generated using ArcGIS 10.2 and is intended for illustrative purposes only.

In this study, aspect data for the region were extracted and analyzed using GIS-based processing of the DEM dataset (Fig 9). The spatial distribution of cultural heritage sites along the Qin–Shu Ancient Road demonstrates a strong correlation with slope aspect. Heritage sites are predominantly concentrated on northwest-facing slopes (292.5°–337.5°, 15.20%), southwest-facing slopes (202.5°–247.5°, 14.85%), and west-facing slopes (247.5°–292.5°, 13.64%) (Table 6). These directions provide relatively stable and favorable microenvironments, which facilitated the construction of the ancient road and the establishment of settlements. In contrast, fewer cultural heritage sites are found on east- and north-facing slopes, a pattern likely attributable to reduced solar exposure or colder climatic conditions in these orientations.

**Table 6:** Distribution of Qin–Shu Ancient Road Cultural Heritage by Slope Aspect.

Range/˚	Orientation	Number/unit	Proportion/%
0–22.5	North	69	11.92
22.5–67.5	Northeast	67	11.57
67.5–112.5	East	38	6.56
112.5–157.5	Southeast	42	7.25
157.5–202.5	South	70	12.09
202.5–247.5	Southwest	86	14.85
247.5–292.5	West	79	13.64
292.5–337.5	Northwest	88	15.20
337.5–360	North	40	6.91

(5) **Precipitation**

**Fig 10:**
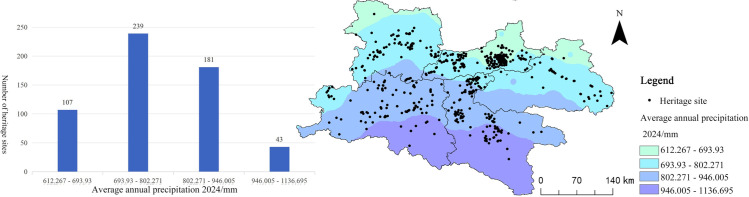
Relationship between Qin–Shu Ancient Road Cultural Heritage and Precipitation. This figure was generated using ArcGIS 10.2 and is intended for illustrative purposes only.

Precipitation is a significant factor influencing human settlement patterns, agricultural productivity, and the spatial distribution of cultural heritage. In this study, spatial interpolation of meteorological station data was performed in a GIS environment to create regional precipitation maps, which were subsequently overlaid with the spatial distribution of heritage sites (Fig 10). The analysis reveals that regions with annual precipitation between 600 and 800 mm encompass the highest density of cultural heritage sites, with 41.62% of the total heritage sites situated within this range. This level of precipitation provides abundant water resources for agriculture and offers an optimal natural environment for human habitation, thereby facilitating the flourishing of cultural activities. In comparison, areas receiving less than 600 mm or more than 1000 mm of precipitation have considerably lower heritage site densities. These extreme precipitation ranges often result in either water scarcity or excessive moisture, which can lead to soil erosion or overly humid conditions, both of which inhibit the accumulation and preservation of cultural heritage.

(6) **Temperature**

**Fig 11:**
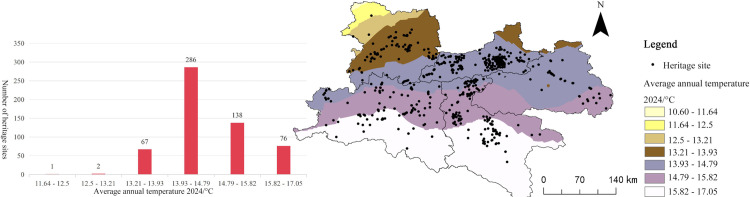
Relationship between Qin–Shu Ancient Road Cultural Heritage and Temperature. This figure was generated using ArcGIS 10.2 and is intended for illustrative purposes only.

Temperature is another crucial climatic variable that affects both human settlement and the distribution of cultural heritage sites. Average annual temperature data were extracted and spatially analyzed in a GIS framework to examine their association with heritage site distribution (Fig 11). The histogram of annual mean temperature and the number of heritage sites demonstrates a marked preference for specific temperature ranges. A majority of heritage sites (n = 286, accounting for 76.34% of the total) are concentrated in areas with average annual temperatures ranging from 13.93°C to 14.79°C. This temperature interval provides optimal conditions for agricultural activities and sustained human habitation, which supports the development of transportation corridors and the persistence of cultural infrastructure. In contrast, regions where the mean annual temperature is below 12.5°C or above 15.82°C exhibit a marked reduction in heritage site numbers, as such extremes restrict the sustainability of settlements and the continuous evolution of cultural activities.

(7) **Soil Type**

**Fig 12:**
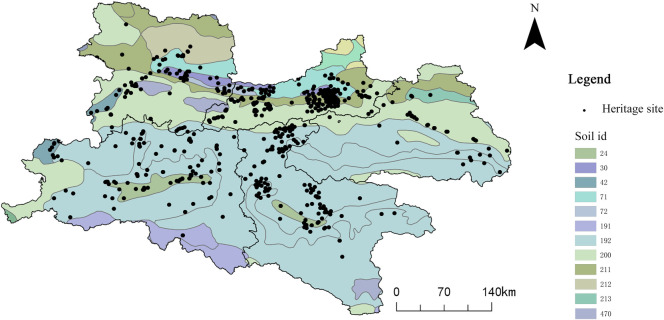
Relationship between Qin–Shu Ancient Road Cultural Heritage and Soil Types. This figure was generated using ArcGIS 10.2 and is intended for illustrative purposes only.

Soil type is a key environmental parameter influencing agricultural production and the spatial distribution of cultural heritage. Spatial overlay analysis using soil classification maps and heritage site locations was conducted within a GIS environment to clarify their relationship (Fig 12 and Table 7). The results indicate that areas characterized by clay pan yellow-brown soil (210 heritage sites, 56.07% of the total) and brown soil (118 heritage sites, 31.51%) contain the highest densities of cultural heritage sites. These soil types possess high fertility and favorable conditions for agriculture, providing a foundation for the emergence and development of ancient cultural activities. In contrast, heritage sites located in regions dominated by less fertile soils, such as alpine shrub meadow soil (n = 6, 1.60%), are much less common, as poor soil quality and suboptimal agricultural conditions hinder both human settlement and the development of cultural activities.

**Table 7:** Distribution of Qin–Shu Ancient Road Cultural Heritage by Soil Type.

Serial Number	Soil ID	Soil Name	Quantity
1	192	Clay Plate Yellow-Brown Soil	210
2	200	Brown Soil	118
3	211	Chestnut Soil	83
4	30	Northern Rice Paddy Soil	53
5	71	Oil Loam Soil	36
6	24	Ma Gan Ni Tian Soil	32
7	42	Yellow-Jiang Soil	12
8	72	Stubble Loam Soil	9
9	470	Mountain Shrubland Meadow Soil	6
10	212	Leached Chestnut Soil	5
11	213	Carbonate Chestnut Soil	3
12	191	Yellow-Brown Soil	2

#### 3.3.2. Human influence factors.

(1) **Ancient transportation**

**Fig 13:**
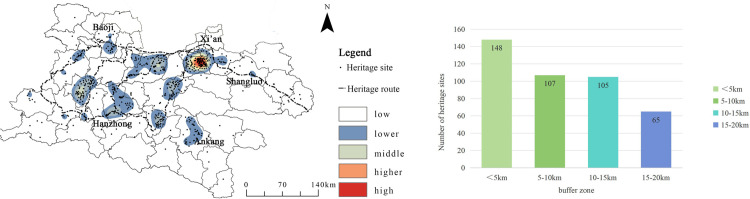
The Relationship between Qin-Shu Ancient Road heritage and traffic. This figure was generated using ArcGIS 10.2 and is intended for illustrative purposes only.

To study the relationship between the Qin–Shu Ancient Road heritage resources and the ancient road itself, the Qin–Shu Ancient Road was overlaid with the kernel density map on the heritage resource distribution layer (Fig 13). A 5 km buffer zone was set around the road. The results show that 73.4% of the heritage sites are located within a 20 km buffer zone of the ancient road (425 sites), with the 10 km buffer zone accounting for 44% (255 sites). This indicates that the distribution of heritage sites is highly dependent on the historical transportation network. Additionally, cities such as Xi’an (190 sites), Mianxian (58 sites), and Chenggu (42 sites), which served as transportation hubs and military strongholds along the ancient roads, exhibit a heritage density (>5 sites/100 km^2^) far exceeding that of surrounding areas (<1 site/100 km^2^). This further validates the “roads foster cities, and cities concentrate heritage” coupling mechanism.

(2) **City GDP**

**Fig 14:**
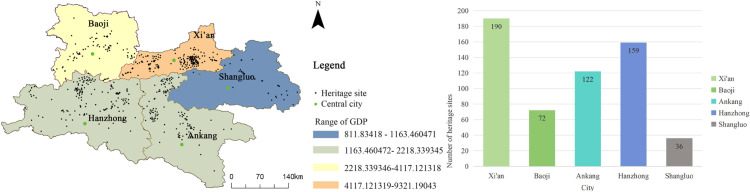
Relationship between Qin-Shu Ancient Road heritage and urban GDP. This figure was generated using ArcGIS 10.2 and is intended for illustrative purposes only.

Based on the overlay of urban GDP and heritage site distributions in Fig 14, Xi’an—serving as the political, cultural, and transportation nexus of the Guanzhong Plain—accounts for 32.8% of all Qin–Shu Ancient Road sites, underpinned by 38.3% of Shaanxi’s GDP. Hanzhong ranks second, where heritage site counts scale proportionally with regional economic output. Ankang and Baoji, both in the province’s mid-economic tier, each sustain a relatively balanced share of relic concentrations, whereas Shangluo, with the lowest GDP contribution, hosts the fewest sites. This spatial pattern demonstrates that local governmental and societal capacities for heritage survey, conservation, and adaptive reuse are tightly constrained by the underlying economic base and available fiscal investment.

(3) **Ethnic Culture**

The Qin-Shu Ancient Road in Shaanxi is a historical crossroads where diverse ethnic cultures have merged. It has served as a hub for multiple ethnic groups, including the Han, Hui, Tibetan, and other minority communities. The interactions between these ethnic groups, driven by trade, migration, and religious activities, facilitated extensive cultural exchanges. The fusion of different ethnic cultures has not only influenced the tangible aspects of cultural heritage but has also shaped intangible elements such as language, rituals, and festivals, enriching the region’s cultural heritage. This integration and interaction of ethnic cultures have added unique diversity and complexity to the spatial-temporal distribution of cultural heritage in the area.

#### 3.3.2. Multivariate analysis of the influencing factors.

This study employs the spatial distribution density of cultural heritage sites along the Qin-Shu Ancient Road in Shaanxi as the dependent variable (Y), and selects multiple key driving factors as independent variables (X), encompassing natural geographical factors—such as altitude, slope, aspect, temperature, precipitation, and soil type—and anthropogenic factors, including ancient road routes and socio-economic conditions (Table 8). The Geographic Detector model is utilized to quantify each variable’s explanatory power regarding the spatial distribution of cultural heritage.

**Table 8:** The Impact of Different Indicators on the Spatial Distribution of the Qin-Shu Ancient Road Cultural Heritage in Shaanxi.

Serial Number	Detection Factor	Index Factors	q statistic	p value
x1	Altitude	Altitude elevation/ m	0.85	0.001
x2	River	River distance/ km	0.55	0.003
x3	Soil Type	Soil Type	0.4	0.05
x4	Transportation	Transportation	0.5	0.003
x5	Gross Domestic Product	GDP/ yuan	0.12	0.027
x6	Slope	Degree/ °	0.52	0.002
x7	Aspect	Orientation	0.43	0.002
x8	Precipitation	Average annual precipitation/ mm	0.73	0.003
x9	Temperature	Average annual temperature/ °C	0.19	0.12

The results indicate that the primary factors influencing the spatial distribution of Qin–Shu Ancient Road heritage sites, in order of explanatory power, are ranked as follows: altitude > precipitation > river proximity > slope > ancient road routes > aspect > soil type > temperature > GDP. Altitude exerts a significant influence on regional climate and ecological environments, while precipitation directly affects the preservation conditions of heritage sites, particularly in humid areas. Rivers demonstrate a pronounced clustering effect around water sources, providing essential natural conditions for heritage preservation. Slope impacts heritage exposure and the risk of natural disasters. The ancient road routes exhibit considerable explanatory power, reflecting the critical role of accessibility in cultural transmission. Although soil type and aspect show relatively lower q-values, both attain statistical significance, suggesting their auxiliary role in shaping spatial patterns. GDP has minimal impact, and temperature exhibits an insignificant effect due to minimal fluctuation in regional temperature patterns.

**Fig 15:**
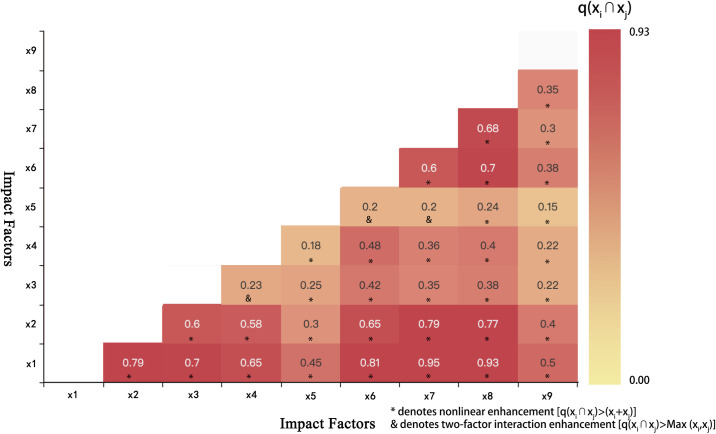
Interaction Effects of Influencing Factors on the Spatial Distribution of Cultural Heritage Sites along the Qin–Shu Ancient Road in Shaanxi.

Interaction analysis reveals that the spatial distribution of Qin–Shu Ancient Road cultural heritage is predominantly driven by the combined effects of natural geographical factors. Notably, significant nonlinear enhancement effects between altitude, aspect, and precipitation underscore the synergistic role of topography and microclimatic conditions in structuring heritage patterns (Fig 15). Interactions involving river proximity with aspect and precipitation further highlight the critical influence of hydrological environments on heritage clustering and preservation. Conversely, interactions between soil type, ancient road routes, and temperature are comparatively weak, reflecting limitations in the coupling between anthropogenic and natural variables. Overall, spatial heterogeneity in natural conditions, mediated by complex interactive mechanisms, profoundly shapes the spatiotemporal distribution of cultural heritage, emphasizing the need for integrated consideration of multiple factors in heritage conservation and regional planning.

## 4. Conclusion and discussion

### 4.1. Discussion

#### 4.1.1. Rigid constraints of natural geography and the spatial logic of linear cultural heritage.

The spatial distribution of cultural heritage along the Qin–Shu Ancient Road is stringently governed by natural geographical factors, most notably elevation, precipitation, and slope. This pattern aligns with the globally observed “dual terrain–hydrology driving” mechanism in linear heritage studies [9]. Yet, compared to the Silk Road [10] and the Inca Trail [13], the Qin–Shu corridor exhibits an even stronger dependence on natural conditions: heritage sites cluster predominantly in low-elevation plains and river valleys—preferentially situated on moderate slopes adjacent to watercourses; form secondary clusters in mid-mountain zones, typically comprising traditional villages or relay stations; and occur sparsely in high-mountain areas, confined to strategic military passes. These observations corroborate Verhagen & Jeneson’s “passive adaptation pathway” theory [8] and substantiate the Chinese mountain-road wisdom of “water determines the course, terrain establishes the route” in mountain-type linear heritage construction [33].

#### 4.1.2. Threshold effects of socioeconomic factors and spatial reconstruction.

While natural geographic factors fundamentally govern the spatial distribution of cultural heritage along the Qin–Shu Ancient Road, anthropogenic drivers—including buffer distance to the route, regional economic development (GDP), and the corridor’s multicultural legacy—exert a nonlinear co-regulatory influence. Spatial analysis demonstrates that the coupling coefficient between heritage sites and the ancient alignment peaks at 73.4% within a 20 km buffer zone and then declines sharply, closely mirroring Tweed & Sutherland’s “linear decay model of heritage dependence” [49] and revealing a pronounced threshold effect. Moreover, high-GDP areas, bolstered by economic expansion, enhanced transportation infrastructure, and concentrated administrative support, have stimulated both heritage redevelopment and preservation investment, creating discernible clustering of sites. Layered atop these dynamics is the historic convergence of Han, Hui, Tibetan, and other ethnic groups, whose intercultural exchanges enrich the temporal and spatial complexity of the region’s cultural heritage.

#### 4.1.3. Theoretical expansion of the temporal-spatial evolution mechanism of cultural heritage.

The evolution of cultural heritage along the Qin–Shu Ancient Road follows a three-stage model—”natural selection – technological breakthrough – cultural adaptation”—illuminating the spatial reconfiguration of cultural routes and dynamic landscapes:

1. Nature-Dominated Phase (Prehistory–Qin–Han): Under strict ecological constraints, site selection depended on proximity to watercourses and low elevations. Heritage sites formed linear settlements along the Wei River and its tributaries, reflecting an “adaptation to terrain and hydrology” strategy [33].
2. Technology-Driven Phase (Wei–Jin to Sui–Tang): Advances in suspension-bridge and plank-road engineering overcame topographic barriers, extending heritage distribution into mountainous regions. By the Sui–Tang–Five Dynasties, the number of sites had reached 311, dominated by cultural landscapes and religious remains, paralleling the Inca Trail’s high-altitude stone-road expansion [13].
3. Cultural-Reconstruction Phase (Yuan–Qing): The road’s role evolved from a transport corridor to multifaceted cultural landscape, with 579 recorded sites, including numerous traditional villages and 37 burial complexes. This shift aligns with dynamic heritage landscape theory [12] and responded to the Song–Ming economic center’s 83.6 km southwestward shift, demonstrating the adaptive realignment of cultural routes to socio-economic change.

### 4.2. Practical Implications

Based on the temporal-spatial pattern evolution principles and driving mechanisms revealed by the study results, a strategic system for the protection of the Qin-Shu Ancient Road is proposed:

1 Integration of Cultural Heritage through Ecological Corridors

The Qin–Shu Ancient Road corridor contains 579 cultural heritage sites, exhibiting a distinct “line–belt–core” spatial configuration. Primary concentrations are found in Xi’an (181 sites), Hanzhong (159 sites), and Ankang (122 sites), with 86.2% of all sites situated below 1,000 m in elevation. To safeguard these resources—particularly those located on yellow-brown loess and brown soils (210 and 118 sites, respectively)—we propose establishing interconnected ecological corridors within low-elevation river valleys linking the Xi’an–Hanzhong–Ankang core zones. These corridors should integrate existing riparian woodlands, wetlands, and agricultural landscapes to conserve both soil and hydrological integrity while providing immersive natural–cultural experiences for visitors.

2 Designing a “Heritage Trail–Cultural Station” Network

Spatial analysis indicates a high density of heritage remains along the Xi’an–Baoji–Hanzhong axis, with technological innovations historically enabling site expansion from valley floors into the Qinling Mountains. Accordingly, we recommend planning a continuous 200–300 km Heritage Trail along this main axis, punctuated by Cultural Stations near secondary cores such as Baoji and Shangluo and adjacent to 135 key sites within a 3 km buffer of major waterways. Each station (200–500 m^2^) should be located in or near traditional villages or site clusters, equipped with digital interpretation tools (e.g., AR/VR) and interactive exhibits, and connected to local transport and educational tour offerings. This approach would transform the former transport corridor into a living cultural–landscape experience.

3 Participatory Community Co-Management

Given that 73.4% of heritage sites lie within a 20 km corridor of the ancient route and closely align with regions of higher GDP, a stakeholder-driven governance model is essential. In economically active areas such as Xi’an and Hanzhong, we advocate convening multi-stakeholder workshops—including government agencies, cultural authorities, tourism operators, and local community representatives—to co-develop conservation and activation plans. Concurrently, training programs on traditional crafts and heritage interpretation should be implemented to form local community volunteer teams. A transparent revenue-sharing mechanism and support for community-led cultural events, thematic homestays, and artisanal cooperatives will ensure that preservation efforts yield tangible local benefits under a “shared responsibility, shared development, shared returns” framework.

### 4.3. Conclusions

This study systematically reveals the temporal-spatial distribution patterns and driving mechanisms of the Qin-Shu Ancient Road cultural heritage through multi-dimensional spatial analysis methods. The main conclusions are as follows:

1. The 579 cultural heritage sites along the Qin–Shu Ancient Road exhibit a distinct “line–belt–core” pattern. The ancient route forms a corridor characterized by belt-like aggregations, with core concentrations located in Xi’an (181 sites), Hanzhong (159 sites), and Ankang (122 sites). Baoji (72 sites) and Shangluo (36 sites) are secondary core zones, collectively covering 32 counties. By site type, ancient road relics predominantly cluster along the Xi’an–Baoji–Hanzhong axis. Traditional villages predominantly occupy valley floors and gentle slopes. Historic architecture is concentrated in Xi’an. Cave temples and stone carvings are situated primarily on cliff faces. Modern historical sites continue to cluster along the transportation corridor. Ancient tombs display a dispersed belt-like distribution influenced by funerary customs and topography.
2. The spatiotemporal evolution of Qin–Shu Ancient Road cultural heritage follows a three-stage model of “natural selection to technological breakthrough to cultural adaptation.” During the nature-dominated phase (Prehistory to Qin–Han), heritage sites were strictly confined to low-elevation river valleys, forming a linear pattern along the Wei River and its tributaries. In the technology-driven phase (Wei–Jin to Sui–Tang), advances in plank road construction facilitated the expansion of heritage sites into the Qinling Mountains, demonstrating the capacity of engineering innovations to overcome natural constraints. During the cultural reconstruction phase (Song to Qing), the heritage sites shifted from transportation infrastructure to settlements and tomb complexes, reflecting a redefinition of the linear cultural heritage’s functional value—from a “transport corridor” to a “cultural landscape.”
3. The primary factors governing the distribution of Qin–Shu Ancient Road heritage sites are elevation, precipitation, slope, aspect, and proximity to hydrological networks, while secondary anthropogenic factors, such as ancient route alignment and regional GDP, exert a comparatively minor influence. Interaction analyses reveal significant synergistic effects between elevation and aspect, as well as between elevation and precipitation. Interactions involving rivers with aspect and precipitation further underscore the pivotal role of hydrological environments in the aggregation and preservation of heritage sites. From a physical geographical perspective, 86.2% of heritage sites are located below 1,000 meters in elevation; the majority of sites are found on yellow-brown loess soils (210 sites) and brown soils (118 sites); 41.6% of sites lie within areas receiving 600–800 mm of annual precipitation; 176 sites are located on slopes between 10° and 20°; aspects facing northwest, southwest, and due west collectively account for over 43%; approximately 82% of sites are situated within a 3-kilometer buffer zone of major waterways; and 43.4% fall within the mean annual temperature range of 13.93–14.79°C. From a cultural perspective, 73.4% of heritage sites lie within a 20-kilometer corridor of the ancient route, with a pronounced concentration in economically developed areas.

## Supporting information

S1 Dataset Heritage Sites - Coordinates Dataset. (XLS)

## References

[1] Bleibleh S, Awad J. Preserving cultural heritage: shifting paradigms in the face of war, occupation, and identity. J Cult Herit. 2020;44:196–203.

[2] Zhang J, Huo XW. Building a new pattern of space protection and utilization of cultural heritage. China J Nat Res. 2019;:005.

[3] Luo L. Research on spatial differentiation of tourism resources and tourism development in Great Road of Sichuan province from the perspective of heritage corridor. Chengdu Univ Technol. 2020.

[4] Huang SZ. A historical study of the transportation routes connecting Shensi and Szechuan provinces. Acta Geograp Sin. 1959;23:419–35.

[5] Li JC. The historical context of the rise and fall of Shu Road traffic. J Sanmenxia Polytech. 2014;13:6–12.

[6] Sun H. Preliminary discussion on the Shu road heritage—dates, route and heritage type. Res Herit Pres. 2017;2:1–9.

[7] Li W, Jiao J, Qi J, Ma Y. The spatial and temporal differentiation characteristics of cultural heritage in the Yellow River Basin. PLoS One. 2022;17(6):e0268921. doi: 10.1371/journal.pone.0268921 35679336 PMC9182564

[8] Verhagen P, Jeneson K. A Roman puzzle: Trying to find the Via Belgica with GIS. J Archaeol Sci. 2012;39(7):2301. doi: 10.1016/j.jas.2012.03.002

[9] Williams T. The Silk Roads: An ICOMOS Thematic Study. ICOMOS; 2017.

[10] Sun H, Zhao W, Li J. Temporal and spatial evolution of the Silk Road cultural heritage and its influencing factors. Hist Geogr Res. 2018;24(2):55–72.

[11] Zhao J, Xu J, Wang G. Analysis of the temporal and spatial pattern evolution of cultural heritage in the Yellow River Basin. Geograph Res. 2020;39(3):478–90.

[12] Fairclough G. Cultural landscape challenges in Europe. J Cult Herit Manag Sustain Dev. 2018;8(3):245–59. doi: 10.1108/JCHMSD-01-2018-0004

[13] Zapata J, Ramirez P, Gomez M. The Qhapaq Ñan: A cultural landscape approach for the conservation of the Inca road system. Conserv Manag Archaeol Site. 2020;22(1):67–85. doi: 10.1080/13505033.2020.1712193

[14] Xu X, Zhang J, Liu S, Liu J, Zhang Z, Tian X. Entropy Change of Historical and Cultural Heritage in Traditional Tibetan Area of China Based on Spatial-Temporal Distribution Pattern. Buildings. 2023;13(12):2995. doi: 10.3390/buildings13122995

[15] Pang L, Wu L. Distribution characteristics and influencing factors of Intangible Cultural Heritage in Beijing-Tianjin-Hebei. Herit Sci. 2023;11(1). doi: 10.1186/s40494-023-00862-2

[16] Capello R, Nijkamp P. Heritage and regional development: The role of linear cultural landscapes. Reg Stud. 2019;53(5):718–32. doi: 10.1080/00343404.2019.1566703

[17] He L. Changes and causes of the traffic roads from Hanzhong basin to Guanzhong plain in Han dynasty. J Shaanxi Univ Technol (Soc Sci). 2008;26:87–91.

[18] Wang ZJ. The management of the roads to Sichuan by people in Qin State. J Xianyang Norm Uni. 2012;27:7–11.

[19] Sun QX. History of the three kingdoms and Shu path. Shaanxi Arch. 2016;01:22–5.

[20] Li ZQ. On the special position of the old way in each posthouse in Sichuan and Shaanxi. Chin Hist Geogr. 1993;2:151–70.

[21] Li JC. Research on Early History of Gu Royte. J Shangqiu Voc Tech Coll. 2016;15:103-106–9.

[22] Dang Y. The development, change and historical function of Baoxie Road. J Tangdu. 1997;4:76–9.

[23] Wang YP, Xu GT, Gao T, et al. Archaeological investigation from Luogu road to the ancient Tangluo road in the Qinling mountains. Relics Museol. 2017;03:18–26.

[24] Li ZQ. The ziwu road in history. J Northwest Uni (Philos Soc Sci Edit). 1981;02:38–41.

[25] Editorial Committee of Shaanxi Ancient Qin-Shu Roads Heritage. Shaanxi Ancient Qin-Shu Roads Heritage. Sanqin Press; 2015:1–10.

[26] Yan Z. Historical and geographical research of Qinshu ancient road. Science Press; 2015.

[27] Chen YY. The ancient Shu road based on the “trinity” of the linear cultural heritage protected mode—Jianmenshudao centered. J Chinese Cult. 2014;02:73–9.

[28] Zhao XN, Guo Y. Situations and approaches of Shudao (Sichuan section) researches in the perspective of cultural route theory. J Southwest Jiaotong Uni (Soc Sci). 2015;:32–9.

[29] Tang F. A rustic opinion of research & protection of Shu Road heritage. Stud Nat Cult Herit. 2017;02:10–9.

[30] Shang CW. Interpretation and utilization conception of Jinniu Dao as linear cultural heritage. Stud Nat Cult Herit. 2017;2:20–9.

[31] Feng MY, Tang GH, Li QY. Research of the tourist value of the ancient Shu Road. J China W Norm Univ (Nat Sci). 2007;04:361–4.

[32] Li YP. Shu road poetry and the development of Shu road tourism resources. J Shaanxi Uni Technol (Soc Sci). 2016;03:49–54.

[33] Li Z. On the nominal problem of Baoxiedao. J Chengdu Univ (Soc Sci Ed). 1989;(1):8-19.

[34] Peng T. Research on the value and heritage composition of the Qinling section of the Qin-Shu Ancient Road from the perspective of cultural routes. Xi’an University of Architecture and Technology; 2022.

[35] Yue J, Dai X. Spatial-temporal pattern of cultural heritage in Beijing-Tianjin-Hebei region and its influencing factors - Taking cultural relics protection units as an example. Econ Geograp. 2020;40(12):221–30.

[36] Huang Y, Xue Q. Spatio-Temporal distribution characteristics and driving factors of traditional villages in the Yellow River Basin. PLoS One. 2024;19(5):e0303396. doi: 10.1371/journal.pone.0303396 38771883 PMC11108216

[37] Zhang Y, Zhang L, Wang J, Dong G, Wei Y. Quantitative analysis of NDVI driving factors based on the geographical detector model in the Chengdu-Chongqing region, China. Ecol Indic. 2023;155:110978. doi: 10.1016/j.ecolind.2023.110978

[38] Wang W, Yang Y. Spatial-temporal differentiation characteristics and driving factors of China’s energy eco-efficiency based on geographical detector model. J Clean Prod. 2024;434:140153. doi: 10.1016/j.jclepro.2023.140153

[39] Mu SL, Yuan ZH, Wuritaoketaohu. Spatial differentiation pattern and impacting mechanism of intangible cultural heritages in the Yellow River Basin. J Des Res. 2022;42(06):255–65. doi: 10.7522/j.issn.1000-694X.2022.00090

[40] Li J. The historical context of the rise and fall of Shu Road traffic. J Sanmen Polytech. 2014;13(02):6–12.

[41] Li J. Research on the early history of Gudao. J Shangqiu Voc Tech Coll. 2016;15(04):103–6.

[42] Peng BB. A new exploration of the origin of the ancient road. Baoji Soc Sci. 2012;(3):43–6.

[43] Li J. Research on the early history of Taurus Road. J Chengdu Normal Univ. 2016;32(08):61–5.

[44] Li J. The traffic and its function of Lianyun stack in Yuan, Ming and Qing dynasties. J Xi’an Univ Arts Sci (Soc Sci Edit). 2017;20(01):38–44.

[45] Huang Y, Yang S. Spatio-temporal evolution and distribution of cultural heritage sites along the Suzhou canal of China. Herit Sci. 2023;11(1). doi: 10.1186/s40494-023-01034-y

[46] Gao S, Wang J, Liu S, Xu X, Liao Y, Zhang Z, et al. Spatio-temporal evolution characteristics and influencing factors of traditional villages in the Qiantang River Basin based on historical geographic information. NPJ Herit Sci. 2025;13(1). doi: 10.1038/s40494-025-01665-3

[47] Zhengchen R, Liu J, Ren J, Zhang S, Liu B. Spatio-Temporal Distribution Evolution Characteristics and Geographical Influencing Factors of Cultural Heritage Sites in Xinjiang, China. Land. 2025;14(5):974. doi: 10.3390/land14050974

[48] Gao J, Wang J, Wang Q, Cao Y. Spatio-Temporal Distribution Characteristics of Buddhist Temples and Pagodas in the Liaoning Region, China. Buildings. 2024;14(9):2765. doi: 10.3390/buildings14092765

[49] Tweed C, Sutherland M. Built cultural heritage and sustainable urban development. Landsc Urban Plann. 2007;83(1):62–9. doi: 10.1016/j.landurbplan.2007.05.008

